# Outcome-Based Assessment of the Payment for Mountain Agriculture: A Community-Based Approach to Countering Land Abandonment in Japan

**DOI:** 10.1007/s00267-021-01497-4

**Published:** 2021-07-07

**Authors:** Kikuko Shoyama, Maiko Nishi, Shizuka Hashimoto, Osamu Saito

**Affiliations:** 1 National Research Institute for Earth Science and Disaster Resilience, Tsukuba, Japan; 2 United Nations University Institute for the Advanced Study of Sustainability, Tokyo, Japan; 3 Graduate School of Agriculture and Life Sciences, The University of Tokyo, Tokyo, Japan; 4 Natural Resources and Ecosystem Services, Institute for Global Environmental Strategies, Hayama, Japan

**Keywords:** Community-based PES, Collective management, Farmland abandonment, Farmland liquidation, Community function

## Abstract

Agricultural land accounts for 37% of the world’s terrestrial area, and the multiple functions of agroecosystems—providing food, soil and water retention, and various cultural services—are of great importance for sustainable land management. To ensure that multifunctionality, payment for ecosystem services (PES) schemes have been developed for heterogeneous agroecosystems. However, the effects of the schemes have not been fully measured because, in most cases, they have been implemented as action-oriented programs rather than outcome-based payments. This study examines the effect of a community-based PES (CB-PES) program on the prevention of farmland abandonment to assess the agricultural outcomes of PES implementation in hilly and mountainous areas in Japan. We interviewed farmers in enrolled communities, mapped enrolled plots, and analyzed agricultural census data on the socioeconomic characteristics and farmland management conditions of 12,261 farmers in 960 agricultural communities in a typical hilly and mountainous area of Noto Peninsula in northern Japan. The results confirm that direct payments are effective in enhancing community management and in preventing additional farmland abandonment. In addition, we found that several socioeconomic and environmental factors at both the community and farmer levels—including geographical conditions, collective management activities, absence of successors, farm scale, and off-farm income dependency—simultaneously affected the farmland abandonment process. Specifically, collective practices within and between communities is a significant factor in preventing farmland abandonment more than collaboration with outsiders. Considering the depopulation and aging of rural communities throughout Japan, intercommunity enrollment could improve the effectiveness of CB-PES by upscaling the current payment scheme to maintain community functions.

## Introduction

Payment for ecosystem services (PES) is used to provide financial incentives for various stakeholders and motivate landowners or managers to implement management practices that will improve the provision of ecosystem services (Jacka et al. 2008). Implementation of PES as environmental policy is often associated with political challenges in addressing the efficiency of the payment schemes (Leimona et al. 2015; Van Noordwijk et al. 2012). Previous studies have investigated a variety of PES characteristics, but the effects of payment schemes have not been fully measured because, in most cases, the schemes were developed as action-oriented programs focusing on the implementation of conservation activities. Action-oriented payment schemes have often appeared to underperform, which has generated increased interest in an outcome-based approach to PES for environmental and agricultural conservation (Lankoski 2016). Examples of outcome-based approaches include evaluations of environmental outcomes (e.g., habitat and indicator species), economic outcomes (e.g., income generation and cost reduction), and social outcomes (e.g., farmers’ behavior and social relationships within a community) (Andeltová et al. 2019; Burton and Schwarz 2013; Hejnowicz et al. 2014; Kleijn et al. 2001).

Agricultural land accounts for 37% of the world’s land area (FAOSTAT 2017), and its multiple functions are of great importance for the sustainable use and management of terrestrial ecosystems. To ensure agricultural multifunctionality, it is crucial to develop PES schemes in accordance with heterogeneous agroecosystems. Agriculture in mountainous areas is particularly essential for food security and environmental sustainability (FAO 2019). PES schemes for mountain agriculture, such as additional income support payments to farmers in specific areas in the EU (European Commission 2011) and the Grain-to-Green Program in China (Liu et al. 2008), have been implemented to facilitate the multifunctional use of farmland, including the provision of food, soil conservation, water retention, and cultural inheritance through historical agricultural activities.

In Japan, historical farmlands in hilly and mountainous areas are widely recognized to not only provide agricultural products, but also to maintain the multifunctionality of the rural landscape, including preservation of secondary natural habitats, carbon storage, soil erosion control, prevention of flood disaster events, and various cultural services (Duraiappah et al. 2012; Hashimoto et al. 2015). However, decreased levels of management and abandonment of farmland resulting from depopulation and an aging population in rural areas are a critical policy concern (Hashimoto et al. 2019). The Direct Payment to Farmers in Hilly and Mountainous Areas program, which specifically targets mountainous agricultural areas, was introduced by the national government in 2000 to maintain these areas’ multiple functions as well as enhance food self-sufficiency (Ministry of Agriculture, Forestry and Fisheries 2019). The program differs from those that offer direct payments to individual farmers, which are common in other developed countries. It is characterized by community-based enrollment, and payments are mainly used for collective works to maintain paddies and crop fields in steeply sloped areas.

PES schemes are often implemented in the context of communal resource management in Asia, Africa, and Latin America (Calvet-Mir et al. 2015). Specifically, community-based PES (CB-PES) schemes, which offer contracts and payments to entire communities, have begun to be investigated in terms of program characteristics and outcomes as compared to those of individual payment schemes. Community participation has been found to be more effective in terms of improving societal outcomes, relative to individual-based programs, by improving community assets and social capital (Brownson et al. 2019) and enhancing the collective stewardship of natural resources (Hayes et al. 2015; Ito et al. 2018). Other studies have identified the importance of institutional context and have shown that communal governance plays an important role in promoting participatory decision-making processes as well as in ensuring the equitable distribution of benefits (Hayes and Murtinho 2018; Hayes et al. 2019). These existing studies highlight the need for additional analysis to clarify which community characteristics influence the outcomes of PES in a communal context.

Japan’s Direct Payment to Farmers in Hilly and Mountainous Areas (a CB-PES program) is expected to prevent further farmland abandonment and maintain the multifunctionality provided by mountainous agricultural areas. Farmland abandonment is also a growing concern in the international community (FAO 2006). In fact, farmland abandonment has grown significantly since the 1950s whereas cropland expansion has slowed around the world (Cramer et al. 2008; Ramankutty et al. 2018). Many studies have revealed its drivers and have shown that abandonment is a complex multi-dimensional process that is interlinked with environmental and socioeconomic conditions at multiple scales (Benayas et al. 2007; Díaz et al. 2011; Khanal and Watanabe 2006; Li and Li 2017; Matsui et al. 2014; Osawa et al. 2016; Su et al. 2018). However, only a few studies have addressed the impacts of land use policy on preventing further farmland abandonment in the context of payment systems (Shin and Kim 2020). Moreover, no studies have examined which characteristics, if any, of local communities and farmers affect farmland abandonment or if the structure of farm management has an effect in the context of CB-PES. It thus remains unclear how CB-PES schemes can be effectively designed to target specific agroecosystems or to provide an appropriate level of benefits. Our aim is to assess the outcomes of the current community-based payment program in Japan. We examine the effects of payments on preventing additional farmland abandonment and also analyzed the characteristics of communities and farmers to determine which characteristics affected the outcomes. The implications of the findings are discussed from land-use policy and management perspectives.

## Background

Over the past decade, Asian population growth has caused unprecedented levels of consumption, and changes in income and consumer preferences have resulted in agricultural expansion in Asia (Critchley and Radstake 2017). Many Asian countries are expected to experience population growth until 2050, at which point the population will begin to decline (United Nations 2017). Japan’s depopulation trend began around 2010, and along with the aging population, land abandonment and underuse of natural resources are now critical concerns threatening the multifunctionality of its land in the provision of various ecosystem services (Government of Japan 2014). The national agricultural census reported that farmland decreased from 60,700 km^2^ (16% of the total land) in 1960 to 43,900 km^2^ (11%) in 2019. In Japan, farmland is considered to be abandoned if it remains uncultivated for at least one year and there are no plans for planting or cropping within the next few years. Currently, such farmland accounts for 4230 km^2^ (Census of Agriculture and Forestry in Japan 2015). Abandoned farmland can positively impact the environment by contributing to the restoration of natural ecosystems (Munroe et al. 2013; Queiroz et al. 2014); however, in some cases, negative consequences involve loss of biodiversity, cultural and aesthetic values (Benayas et al. 2007). In addition, local communities in and around abandoned areas have often suffered from agricultural damage caused by increased populations of deer, monkey, wild boar, and other wildlife. Such negative socioeconomic consequences are also of serious concern (Tsuchiya and Hagihara 2017).

For farmland preservation, a variety of programs have been put in place in recent decades in Japan, including new forms of tenancy arrangements, incentive mechanisms, and educational campaigns. Historically, the mainstay of agricultural land policy has been the Agricultural Land Act (ALA) established in 1952. Building on the post-WWII land reform that dismantled landlordism, the ALA stipulated that “the ownership of agricultural land by cultivators themselves is most appropriate” and thus restricted farmland transactions except for acquisition by existing cultivators so as to protect small owner-farmers (Honma 2010). Under the Agricultural Basic Act of 1961, which led to modernizing farming, the ALA went through numerous amendments to loosen restrictions and generate economies of scale alongside industrialization and urbanization in the postwar era (Hori 2012). At the same time, new institutions were introduced to facilitate tenancy arrangements for the productive use of farmland. The farmland liquidation policy has promoted the development of a farmland tenancy market where new farmers can enter and contribute to scale expansion of farm management. In particular, tenancy arrangements have been facilitated by introducing government farmland banking launched in 2014, leading to land transfers to expand and aggregate farmland.

Taking advantage of the longstanding self-governing capacity of farming communities, the government has promoted community-based approaches to policy implementation for agricultural programs (Tabata 2005). Such approaches are epitomized by the Japanese payment program. The Direct Payment to Farmers in Hilly and Mountainous Areas (hereafter called direct payment) was introduced in 2000 following the growing recognition of ‘agricultural multifunctionality’ since the 1990s; the concept of agricultural multifunctionality was put into statutory form as the Food, Agriculture and Rural Areas Basic Act of 1999. This scheme is the first incentive payment in Japan specifically designed to maintain and enhance non- economic functions provided by agroecosystems in mountainous area, which are considered disadvantageous to productivity (Sakuyama 2006). Payments are determined, in part, based on the degree of slope of the enrolled farmland. In 2018, the program covered 7933 km^2^ of sloped farmland (about 19% of the total farmland in the country) where farmland management activities by local communities have been supported for the past two decades. Eligible communities apply to the municipal government to join the program, and enrolled communities must engage in a planned contractual activity under the administration of the municipal government for five years. All decisions about the activity plans are made based on discussions within the community. The contents of the activity plan can include community-based agricultural management, training of successors, and measures to stabilize agricultural income (e.g., introduction of new crops, marketing, and secondary processing). If the community fails to meet the contractual criteria (i.e., are unable to maintain the farmland under the payment contract), it needs to fully refund the payment.

The government has invested more than JPY 53,090 million per year; 25,405 communities (18% of total agricultural communities) have received payments to maintain 6643 km^2^ of farmland, covering more than 80% of the targeted farmland (Ministry of Agriculture, Forestry and Fisheries 2019). The enrolled communities have received approximately JPY 2 million per year on average. The payments have been allocated to individual farmers (48% of total payments) or used for collective practices (52%) such as maintaining farm roads, channels, and furrow irrigation; replanting abandoned land; and purchasing farming machinery and fencing. Because some communities had to forgo applying for the program due to a lack of sufficient farm households in the community, the government introduced the intercommunity partnership system in 2015. Under this system, additional payments are given to participants who enter into agreements with more than one community to manage agricultural activities beyond their own community.

## Methods

### Site Description

**Fig. 1:**
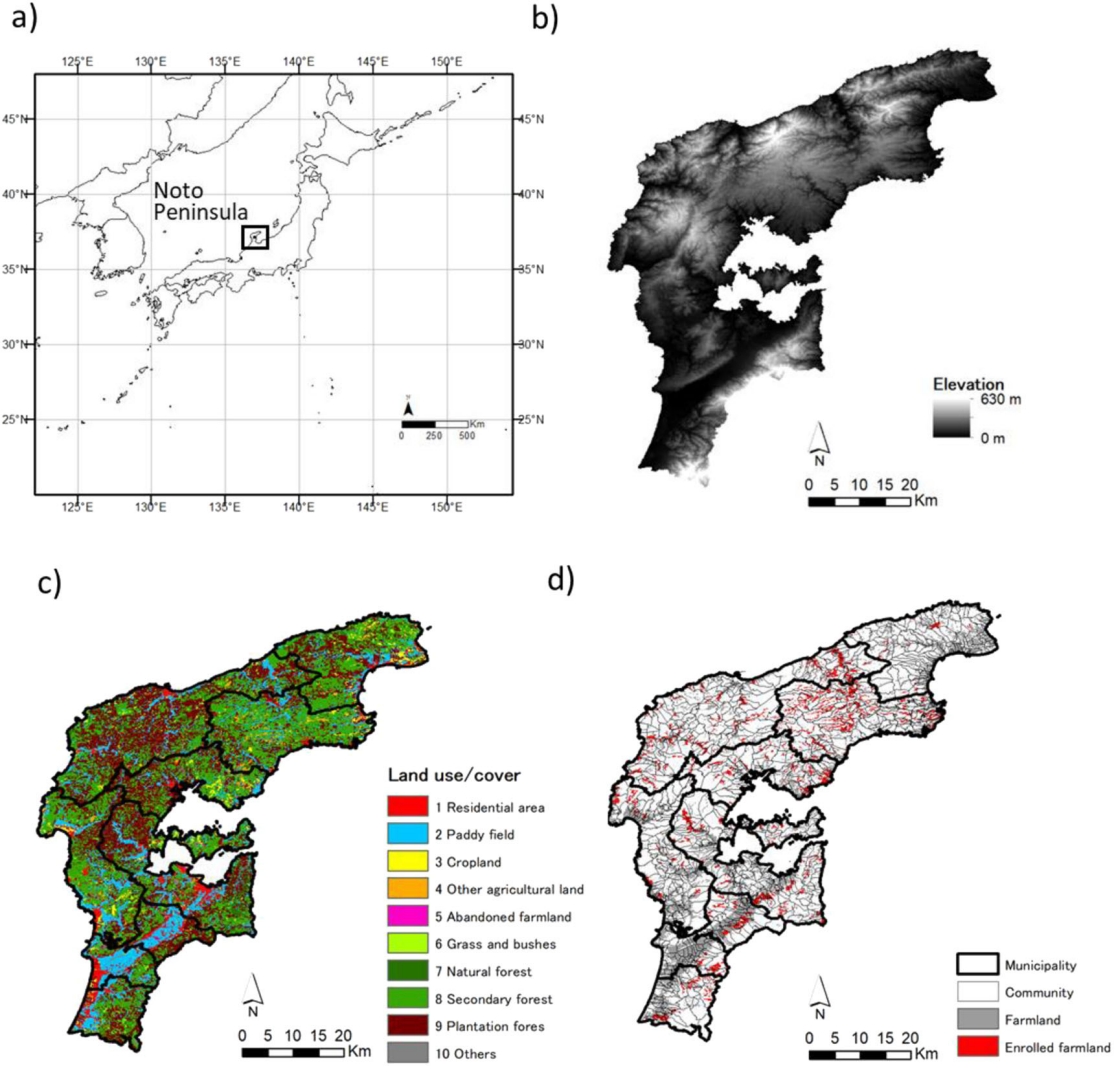
(**a**) Location, (**b**) elevation, (**c**) land use, and (**d**) distribution of enrolled farmland in Noto Peninsula. Data sources: (**b**) elevation model (National Land Numerical Information); (**c**) vegetation map (Biodiversity Center of Japan, Ministry of the Environment); and (**d**) farmland plots (National Chamber of Agriculture of Japan) and enrolled plots (Ishikawa Prefecture)

We selected a typical hilly and mountainous landscape located in the northern part of the Noto Peninsula in Japan as a study site (Fig. 1a). The area has been enrolled in the payment program since it was introduced in 2000. The landscape covers an area of 1978 km^2^ at an elevation ranging from 0 to 629 m (almost half of the area has a steep slope of at least 15°, Fig. 1b) and mainly consists of forest, agricultural and residential area (Fig. 1c). The area has nine local municipalities encompassing a total of 1051 communities and a population of 196,416 in 2015. Farmland extends throughout the entire region, whereas the enrolled plots have a more limited distribution of farmland across different administrative areas (Fig. 1d).

The Census of Agriculture and Forestry in Japan (2015) reported that the total cultivated acreage was 190 km^2^, consisting mostly of paddy fields (153 km^2^) and cropland (12 km^2^). Rice paddy terraces have been developed in steep fields for hundreds of years along the coast and on hillsides and other crops include wheat, beans, and vegetables. In 2011, the region was designated by the UN Food and Agriculture Organization (FAO) as a Globally Important Agricultural Heritage System (GIAHS), with the aim of enhancing sustainable agricultural activities and primary-industry-based tourism in the area. The population of the Noto region has decreased since the mid-1950s, corresponding with national trends. Additionally, out-migration to cities has resulted in the stagnation of agriculture and forestry in the region. The census reported that 8752 residents, accounting for 4.7% of the total population of the region, engaged in agriculture as their main source of livelihood. However, more than 80% of the farmers were over the age of 65, suggesting even more decline in the farming population in the near future.

### Data Collection

To extract potential factors to be included in the analysis, we drew on general insights gained from previous studies and the perspectives of local stakeholders regarding driving factors of farmland abandonment. Previous studies have revealed that environmental factors (e.g., maximum temperature, average precipitation, and geographical conditions) and socioeconomic factors (e.g., farmer type, farm scale, machinery, and labor) affect farmland abandonment in Japan (Matsui et al. 2014; Osawa et al. 2016; Shin and Kim 2020; Su et al. 2018). Thus, we included these environmental and socioeconomic factors in the analysis to examine the payment effects, except for temperature and precipitation because differences of climatic conditions are not significant for agricultural abandonment in the region.

In addition, because our aim is to examine the effects of multilevel factors on abandoned farmland in relation to the direct payments, we examined socioeconomic and farmland management conditions at the community and farmer level. To extract potential factors to be included in our analysis, we conducted face-to-face structured interviews with representative farmers from 22 communities enrolled in the direct payment program from July to December 2017. A national agricultural census is conducted every five years to collect data on socioeconomic factors at the community and farmer level; we used the latest census data (collected in 2015) on the socioeconomic characteristics and farmland management conditions of 12,261 farmers in 960 agricultural communities (i.e., all farmers and agricultural communities in the area). The national census includes 105 items about farm management status for each management entity (including organizations and farm households), and 64 items about community management status for each community. All of these items were included in the interviews to evaluate which potential factors should be included in the analysis.

Based on the interview results, we selected key socioeconomic factors to be used in the statistical analysis: (1) community characteristics: collective practices, including natural resource conservation (e.g., community-based management of forests, agricultural land, ponds, and irrigation channels) and local revitalization activities (e.g., traditional festivals and events, welfare and tourism activities, and renewable energy management) within a community, among multiple communities, and in collaboration with outsiders (e.g., city residents, schools, and non-profit organizations); and land characteristics, including slope and distance to public institutions, community centers, and other buildings and the area of farmland plots enrolled in the payment program in the community; and (2) farmer characteristics: total area of owned and managed farmland, off-farm income dependency, gender and age of household head, existence of a successor, number of hired laborers, farm machinery, crop types, and sales of agricultural products.

The geospatial data including a 10-m digital elevation model and point data of public buildings (source: National Land Numerical Information) were used to calculate the geographical conditions of the land. The distribution map of enrolled plots in Ishikawa Prefecture (2018) was converted to a GIS-based map and calculated the area of each payment plot. Spatial calculations were carried out with ArcGIS ver. 10.7.

### Data Analysis

To examine the extent to which the payment program and key socioeconomic factors impacted land abandonment, we developed a multilevel statistical model. In the model, the dependent variable is the ratio of abandoned farmland (i.e., land that a farmer did not intend to use anymore) to total farmer-owned farmland (data source; Census of Agriculture and Forestry in Japan 2015), and the independent variables are factors selected at the community and farmer levels. We used a generalized linear mixed model, and to account for the effects of individual farmers and communities, we entered a community ID (CID) and a farmer ID (FID) as random effects. The full model was thus:

$$Y_{ij} = \beta _0 + \beta _1C_i + \beta _2F_j + CID_j + FID_i + \varepsilon _{ij},$$

where *Y* is the ratio of abandoned farmland to total farmer-owned farmland by farmer *i* in community *j*, which follows a gamma distribution, *C*_*i*_ is a variable of community *i* and *F*_*j*_ is a variable of farmer *j*; *β*_*n*_ is the coefficient of variable *n*; and *ε*_*ij*_ is the error term with a normal distribution. We built a community-level model, a farmer-level model, a full model (including both farmer- and community- levels), and a null model, and used the Akaike information criterion (AIC) as the basis for model selection (Burnham and Anderson 2002). Correlation tests were performed to examine fixed effects (Appendix I). Statistical analyses were conducted using lme4 package for R version 3.6.1 (R Foundation for Statistical Computing, Vienna, Austria).

## Results

### Overview of Farmland and Enrolled Plots

Table 1 shows the number of enrolled communities, as well as the area of enrolled plots and abandoned farmland in each municipality; 44% of farmer-owned farmland and 34% of farm communities were enrolled in the area. In municipalities located in the northern part of Noto region, which have lower accessibility to the prefectural capital, a relatively high rate of abandoned farmland (20–30%) was observed (Table 1). Three of the four municipalities in the north showed a high level of enrollment in the payment program, with over eight percent of farmland enrolled. Although the rate varied, municipalities with large rates of abandoned farmland appeared to have more communities enrolled in the payment program in all regions.

**Table 1:** Summary statistics of enrolled farm plots and abandoned farmland in the study area

Region	Municipalities	Number of farm communities		Farmland (km^2^)		Abandoned farmland (km^2^)
			Enrolled communities		Enrolled plots	
Oku-Noto (north)	Wajima	181	100 (55.25)	11.66	9.9 (84.91)	2.43 (20.84)
	Suzu	154	16 (10.39)	10.35	1.07 (10.34)	2.16 (20.87)
	Anamizu	69	32 (46.38)	5.83	4.78 (81.99)	1.92 (32.93)
	Noto	132	77 (58.33)	11.11	9.05 (81.46)	2.24 (20.16)
Naka-Noto (middle)	Nanao	152	50 (32.89)	19.29	7.54 (39.09)	3.71 (19.23)
	Nakanoto	49	14 (28.57)	6.59	3.36 (50.99)	0.41 (6.22)
Kuchi-Noto (south)	Hakui	56	6 (10.71)	11.08	1.76 (15.88)	0.73 (6.59)
	Shika	114	16 (14.04)	15.74	3.31 (21.03)	2.59 (16.45)
	Hodatsu-shimizu	53	18 (33.96)	5.89	2.7 (45.84)	0.52 (8.83)
Total		960	329 (34.27)	97.54	43.47 (44.57)	16.71 (17.13)

Farmland includes farmer-owned plots only. The numbers in parentheses are the percentages of enrolled communities, plots, and abandoned farmland (by area)

Table 2 shows the area of owned and managed (cultivated) farmland by type of farm household. The total area of owned farmland in the study area was 97 km^2^ and the total managed farmland area (either owned or rented) was 140 km^2^; this indicates that a substantial amount of farmland was rented out by other landowners, particularly those who do not engage in farming (i.e., non-farmers). Most of the farmland was cultivated by commercial farm entities including farm organizations, full-time farmers, and part-time farmers. The part-time and full-time farmers mostly managed farmland with a high rate of rented farmland (45% and 49% respectively). Whereas farmland owned by farm organizations covered less than 5% of the total farmland area, farm organizations managed far more land rented from other landowners, which amounted to 86% of their managed land. As for noncommercial (self-sufficient) farmers, they abandoned or leased out the most of land for cultivation, so they only managed the remaining 34% of farmland. Abandoned farmland was owned mostly by self-sufficient farmers, followed by part-time and then full-time farmers (Table 2). In total, abandoned farmland amounted to 17% of the total farmers owned farmland.

**Table 2:** Overview of farmland for each farm household type

Farmer type	Total owned farmland				Rented farmland	Total managed farmland
		Planted farmland (a)	Abandoned farmland	Farmland for lease	(b)	(a) + (b)
	(km^2^)	(km^2^)	(km^2^)	(km^2^)	(km^2^)	(km^2^)
Farm organization	4.41	4.1 (92.97)	0.1 (2.27)	0.21 (4.76)	26.46	30.56
Full-time farmers	21.65	17.12 (79.08)	2.65 (12.24)	1.88 (8.68)	16.59	33.71
Part-time farmers	47.3	36.49 (77.15)	6.68 (14.12)	4.13 (8.73)	30.89	67.37
Self-sufficient farmers	24.18	8.31 (34.37)	7.3 (30.19)	8.57 (35.44)	0.7	9.01
Total	97.54	66.02 (67.69)	16.73 (17.15)	14.79 (15.16)	74.64	140.65

Numbers in parentheses are the percentages of planted farmland, abandoned farmland, or farmland for lease to owned farmland

A farm household is defined as a household engaged in farming and managing 10 ares or more of cultivated land or earning more than JPY 150,000 per year from the sale of agricultural products (Ministry of Agriculture, Forestry and Fisheries 2017). A farm household is classified as commercial if it manages 30 ares or more or earns more than JPY 500,000 per year from the sale of agricultural products. Here, commercial households are classified as either full-time (only farming) or part-time (having other jobs) farmers. Noncommercial households are listed as self-sufficient farmers

### Characteristics of Communities and Farmers

The characteristics of communities and farmers analyzed in our study are summarized in Table 3. Regarding community practices, 85% of the communities engaged in collective activities for conservation and management of natural resources and local revitalization within the year. Furthermore, 68% of communities conducted these activities in collaboration with neighboring communities, whereas only 11% of communities collaborated with those from outside the communities to manage their land.

**Table 3:** Summary of variables used in the analysis

Variables	Description	Mean	SD
Dependent variable:			
Farmland abandonment ratio for each farmer (*n* = 12,261)	Ratio of abandoned farmland area to total owned farmland	0.17	0.24
Independent variables:			
*Characteristics of communities* (*n* = 960)			
Payment	Area of enrolled plots in the community (ha)	4.6	9.2
Community-based activities	0: None (14.6%); 1: Yes (85.4%)	−	−
Cooperation between communities	0: None (31.4%); 1: Yes (68.6%)	−	−
Cooperation with outsiders	0: None (88.6%); 1: Yes (11.4%)	–	−
Slope	Average slope (degrees)	14.4	8.9
Distance to the town center	Average distance to the town center (km)	5.8	4.2
*Characteristics of farmers* (*n* = 12,261)			
Total farmland	Total owned farmland (are)	77.4	80.6
Off-farm income dependency	0: No dependency (18.5%); 1: Partial dependency (40.5%); 2: Full dependency (41.0%)	−	−
Gender	0: Female (3.9%); 1: Male (96.1%)	−	−
Age	Age of household head (years)	69.3	10.1
Successor	0: None (52.8%); 1: Yes (47.2%)	−	−
Labor	Number of hired laborers	0.62	2.2
Machine	Number of farm machines	2.2	1.4
Crop type	Number of crop types	1.6	2.1
Sales	0–15 (annual sales level from <150,000 JPY to 500 million JPY)	2.1	2.2

Data sources: Census of Agriculture and Forestry 2015 for the characteristics of farmers and collective practices. Area of enrolled plots in the community, slope and distance to the town center were calculated using the GIS-based dataset developed in this study

Off-farm income dependency was defined based on the definition of farmer type: 0, farm organization and full-time farmer; 1, part-time farmer; and 2, self-sufficient farmer. Types of farmer are defined in Table 2. The data contains null values in gender, age, and successor for 6893 farmers

The farmers consisted of 2024 full-time farmers, 4962 part-time farmers, 5033 self-sufficient farmers, and 242 farm organizations. Overall, part-time farmers who depend more or less on other income sources and self-sufficient farmers made up about 80% of all farmers. The household heads were mostly male and ranged in age from 26 to 101 years, with a mean age of 69 years. Only 3295 farmers (47% of the commercial farmers) had a successor, while the number of hired laborers ranged from 0–59 (average 0.6), the number of agricultural machines ranged from 0 to 11 (average 2.2), and the number of crop types ranged from 0 to 20 (average 1.6). The average annual sales level ranged from less than 150,000 to 5 hundred million JPY.

### Multilevel Model Estimation

Table 4 presents results derived from the multilevel model. The main objective was to examine the effects of the payment program as well as the effects of various characteristics of communities and farmers on farmland abandonment. The null model (Model 1) had a farmer-level variance of 0.03 and a community-level variance of 0.011, but the community model (Model 2), farmer model (Model 3), and the full model (Model 4) had lower values of variance. AIC values were smaller in order Model 1 > Model 3 > Model 2 > Model 4. On the basis of AIC, the full model was selected as the best-fitting model.

**Table 4:** Multilevel model estimation of the effects of various factors on the abandoned farmland ratio

Variable	Model 1		Model 2		Model 3		Model 4	
	Estimate	SE	Estimate	SE	Estimate	SE	Estimate	SE
Intercept	0.142	0.004^***^	0.133	0.020^***^	0.209	0.022^***^	0.199	0.029^***^
*Characteristics of communities*								
Payment			–0.001	3.946E−04^**^			–9.82E−04	3.887E−04^*^
Community-based activities			–0.073	0.018^***^			−0.070	0.018^***^
Cooperation between communities			–0.019	0.009^*^		−0.017	0.009^*^	
Cooperation with outside			–0.007	0.013			−0.004	0.013
Slope			0.004	0.001^***^			0.004	0.001^***^
Distance to the town center			0.728	0.110^***^			0.711	0.109^***^
*Characteristics of farmers*								
Total farmland					2.157E−04	2.685E−05^***^	2.105E−04	2.666E−05^***^
Off-farm income dependency					0.009	0.005	0.010	0.005^*^
Gender					0.010	0.011	0.009	0.011
Age					–4.093E−04	2.281E−04	–4.232E−04	2.275E−04
Successor					−0.012	0.005^**^	–0.012	0.005^**^
Labor					0.001	0.001	0.001	0.001
Machine					−0.003	0.002	−0.002	0.002
Crop type					−0.005	0.001^***^	−0.005	0.001^***^
Sales					−0.016	0.002^***^	−0.015	0.002^***^
Farmer-level variance	0.030		0.030		0.029		0.029	
Community-level variance	0.011		0.008		0.010		0.008	
Log-likelihood	1837.5		1881.2		1883.2		1920.4	
AICc	–3669.1		–3744.4		–3742.4		–3804.7	
delta-AIC	135.7		60.3		62.3		0.0	
AIC weight	0.00		0.00		0.00		1.00	
df	3		9		12		18	
Number of observations	6893		6893		6893		6893	
Number of groups	836		836		836		836	

A negative number indicates negative effects

*AIC* akaike information criterion

***P < 0.001; **P < 0.01; *P < 0.05

In the multi-level model, results clearly showed a significant payment effect at the community level in both the community model and the full model (*P* < 0.01 and *P* < 0.05, respectively). In terms of collective practices, community-based activities and cooperation between neighbor communities were negatively correlated to the abandoned farmland ratio (*P* < 0.001 and *P* < 0.05, respectively), but collaboration with outsiders was not a significant factor. This suggests that collective practices within and between communities could prevent farmland abandonment more so than collaboration with outsiders. Both slope and distance to the town center were positively correlated with the farm abandonment ratio (*P* < 0.001), indicating that communities with farmland in steep areas and with low accessibility to the town center tended to abandon more land.

At the farmer level, the total area of farmland owned by the farmers had a positive effect (*P* < 0.001), indicating that farmers tended to abandon more farmland if they owned more farmland. The sales level, number of crop types, and existence of successors all had significant negative effects (all at least *P* < 0.01). These results suggest that high sales and more crop types as well as the existence of successors act as deterrents to farm abandonment. The positive effect of off-farm income dependency (in Model 4) indicates that farmers with more off-farm income (i.e., part-time farmers and self-sufficient farmers) tended to abandon more farmland as compared to farm organizations and full-time farmers.

To enable evaluation of selected variables in analysis at community and farmer levels, summary statistics including AICc and AIC weights for the ranked regression models are shown in Appendix II. In the community-level regression models, the best model included the variables: payment, slope, distance to the town center, and community-based activities, which suggests the payment does not affect alone to prevent farmland abandonment (Appendix II-Table 6). For the farmer-level regression models, total farmland, successors, number of crop types, and sales were included in the best model (Appendix II-Table 7). This supports findings from the multi-level model.

## Discussion

### Effective CB-PES on Preventing Farmland Abandonment

We examined the influence of the characteristics of farmers and community practices on farmland abandonment, particularly with regard to the direct payment program. We identified several characteristics that influence farmland abandonment (Table 4). First, enrollment in the current direct payment program was negatively correlated to the abandoned farmland ratio, suggesting the effectiveness of the program. However, farmland abandonment has been increasing for 20 years even under the current payment program. Other socioeconomic and environmental factors at the community- and farmer-levels significantly influenced farmland abandonment, both positively and negatively, as described below.

The farming population throughout the country is aging and decreasing along with the general trends of agricultural downturn, so a shortage of agricultural successors is a primary concern among local communities, and previous studies identified this as a significant factor affecting the abandonment of farmland (Osawa et al. 2016; Shin and Kim 2020; Su et al. 2018). In our models, the age of the head of household was not a significant factor, but the absence of agricultural successors was positively correlated to farm abandonment. The challenging geographical conditions (i.e., steep slopes and low accessibility to the town center) of farmland at the community level also constrained continuous farmland management, as has been reported in previous studies (Khanal and Watanabe 2006; Díaz et al. 2011; Osawa et al. 2016).

In regard to farm management scale, agricultural sales, crop types, and labor status have been found to be significant factors, with varying degrees of influence, depending on the region (Matsui et al. 2014; Shin and Kim 2020; Su et al. 2018). In our models, the sales of agricultural products and the number of crop types per farmer were significant factors in reducing farmland abandonment, but the farmland area was positively related to the abandonment ratio. These results imply that a well-run farm might be of more importance, irrespective of the farming scale. In addition, off-farm income dependency was identified as a significant factor in a model. This suggests that farmers with more off-farm income are more likely to abandon farmlands, perhaps because they find it difficult to maintain the land and are able to rely more on non-farm income sources for their livelihood.

Because abandonment is caused by multiple factors that cannot be addressed by a single policy (Osawa et al. 2016), the payment program should be developed to take these factors into account to strategically define an effective target. Our study confirmed that the fundamental issues, i.e., absence of agricultural successors and challenging geographical conditions are primary factors affecting the abandonment of farmland. In addition, other factors relating to farming scales and farmer types should be considered in designing effective CB-PES. For example, more than 75% of farmers rely more on non-farm income than farm income in the area. The payment program should account for the situation by using a strategy that targets self-sufficient and part-time farmers to participate more in collaboration with full-time farmers and farm organizations within or between communities.

Regarding community practices, our analysis reveals that the enrolled communities engaging in community-based activities are more likely to maintain farmland than non-enrolled and non-collective communities. In addition, cooperation between neighboring communities also contributes to reducing the amount of abandoned farmland. Relatively few communities engaged in rural revitalization activities in collaboration with outsiders, whereas there was a high rate of communities conducting collective practices within or between communities (Table 3). This is because collaboration with outsiders is a still new challenge in regional development whereas the community practices have been historically developed in the region. Existing social cohesiveness facilitates PES participation (Ito et al. 2018); thus, the payment system could be improved by enhancing existing collaboration between neighboring communities.

### Limitations and Future Research

The limitations of this study are related to the limited analysis using a single time-point data. First, this study was conducted using data from both communities that were enrolled and those that were not. A comparative study including information/data from communities before they enrolled into the program, however, would better reveal the effectiveness of the payments. In addition, potential factors that might have influenced community differences among municipalities (such as community cohesiveness, and administrative support by municipalities or associations) were suggested during interviews, but they were not included in the models due to a lack of quantified data as well as qualitative research on these issues. Thus, further research is required to examine multilevel governance, including data at the prefecture and municipality levels.

Second, the direct payment program has been implemented for the past two decades. Because of a lack of successors for aging farmers, however, enrollment into the payment program has gradually decreased over the past 10 years (Ministry of Agriculture, Forestry and Fisheries 2019), especially in depopulated communities. This situation has accelerated farmland abandonment, so the government introduced the intercommunity partnership system in 2015. This new system allows communities to enroll in the direct payment program based on agreements among multiple communities. To date, the system has involved 534 intercommunity agreements and 41,000 ha of enrolled plots throughout the country, a relatively small portion of the total program enrollment (Ministry of Agriculture, Forestry and Fisheries 2019). Additional studies are needed to identify the key factors affecting enrollment into this new system. Incentives and social norms at the neighborhood level have had significant impacts on program enrollment (Chen et al. 2009). However, enhancing intercommunity participation might require increased governance to organize the communities, which may include considering each community’s historical context as well as their socioeconomic and geographic conditions.

Third, from a land-use policy perspective, our findings support the validity of the current farmland liquidation policy to a certain extent. The census data showed that a substantial amount of farmland was rented out by landowners who do not engage in farming (Table 2), e.g., historical landholders, small landowners who inherited their family land, and absentee landowners. The farmland liquidation policy facilitates consolidation and aggregation of farmland for productive land use, but the community-based payment program can compensate for the loss of community functions, which is especially applicable in the hilly and mountainous areas characterized by fragmented farm plots (Nishi 2019). Maintaining the functions of rural communities is fundamental to manage agricultural land and natural resources, and PES should be designed to value collective efforts and promote land stewardship so as to promote agricultural diversity (Kolinjivadi et al. 2019). Further research is required to evaluate these conditions, particularly in relation to the current land-liquidation policy.

## Conclusions

We examined the effects of CB-PES on the ratio of abandoned farmland as well as other factors influencing land abandonment. The simple community-level model showed that payments reduced the amount of abandoned farmland. The farmer-level model, which included farm-level characteristics, explained the phenomena of farmland abandonment in more detail, and the model combining community and farmer characteristics enabled an even more comprehensive understanding of the factors affecting farmland abandonment. A payment effect was observed, but the payment does not affect alone to prevent farmland abandonment. Socioeconomic and environmental factors simultaneously affected the abandonment process within the enrolled agricultural communities. These endogenous and exogenous factors should be further analyzed in connection with land liquidation policy to reform the payment program.

Farmland abandonment is a growing concern in the international community. Considering the negative consequence of depopulation in rural areas, the results from outcome-based assessment could have implications for the effectiveness of CB-PES programs in other countries. Payment systems should be carefully developed and implemented in accordance with societal changes to sustainably maintain the community functions of agriculture in hilly and mountainous areas.

## Appendix I

Table 5

**Table 5:** Correlations among variables used in our models

I. Characteristics of communities variables	Payment	Community-based activities	Cooperation between communities	Cooperation with outsiders	Slope	Distance to the town center
Payment	1.000					
Community-based activities	–0.165	1.000				
Cooperation between communities	0.079	–0.213	1.000			
Cooperation with outsiders	–0.126	–0.053	–0.091	1.000		
Slope	–0.092	0.144	–0.016	–0.025	1.000	
Distance to the town center	–0.061	–0.108	–0.007	0.026	–0.188	1.000

II. Characteristics of farmers	Total farmland	Off-farm income dependency	Gender	Age	Successor	Labor	Machine	Crop type	Sales
Total farmland	1.000								
Off-farm income dependency	0.043	1.000							
Gender	–0.016	0.059	1.000						
Age	0.011	0.313	–0.091	1.000					
Successor	–0.024	–0.212	–0.035	–0.187	1.000				
Labor	–0.013	0.065	–0.050	0.005	–0.010	1.000			
Machine	–0.047	–0.035	0.041	0.025	–0.065	–0.055	1.000		
Crop type	–0.005	0.031	0.025	–0.037	–0.016	–0.019	–0.034	1.000	
Sales	–0.372	0.085	0.059	0.066	–0.035	–0.192	–0.274	–0.083	1.000

## Appendix II

Table 6

**Table 6:** AIC values for the ranked regression models for variation in community-level variables (*N* = 11,925)

Parameters	df	loglik	AICc	delta AIC	AIC weight
pay, slp, dtc, cma	7	1145.0	−2275.9	0.0	0.99
pay, slp, dtc, cma, cmc	8	1141.5	−2266.9	9.0	0.01
pay, slp, dtc, cma, cmc, otc	9	1139.0	−2260.1	15.8	0
pay, slp, dtc	6	1120.8	−2229.5	46.4	0
pay, slp	5	1114.9	−2219.7	56.2	0
pay	4	1076.0	−2144.0	131.9	0

*pay* payment, *slp* slope, *dtc* distance to the town center, *cma* community-based activities, *cmc* cooperation between communities, *otc* cooperation with outsiders

Table 7

**Table 7:** AIC values for the ranked regression models for variation in farmer-level variables (*N* = 6893)

Parameters	df	loglik	AICc	delta AIC	AIC weight
land, scc, crp, sls	7	1904.5	−3794.9	0.0	0.94
land, scc, crp, sls, off_frm	8	1902.6	−3789.2	5.7	0.06
land, scc, crp, sls, off_frm, age, gnd	10	1893.5	−3766.9	28.0	0
land, scc, crp, sls, off_frm, age, gnd, lbr. mcn	12	1883.2	−3742.4	52.5	0
land, scc, crp	5	1832.6	−3655.2	139.7	0
land	4	1067.8	−2127.6	1667.3	0

*land* total farmland, *scc* successor, *crp* crop type, *sls* sales, *off_frm* off-farm income dependency, *age* age, *gen* gender, *lbr* labor, *mcn* machine
