# Correction to: Outcome-based assessment of the payment for mountainagriculture: a community-based approach to countering land abandonment in Japan

**DOI:** 10.1007/s00267-022-01615-w

**Published:** 2022-03-10

**Authors:** Kikuko Shoyama, Maiko Nishi, Shizuka Hashimoto, Osamu Saito

**Affiliations:** 1 National Research Institute for Earth Science and Disaster Resilience, Tsukuba, Japan; 2 United Nations University Institute for the Advanced Study of Sustainability, Tokyo, Japan; 3 Graduate School of Agriculture and Life Sciences, The University of Tokyo, Tokyo, Japan; 4 Natural Resources and Ecosystem Services, Institute for Global Environmental Strategies, Hayama, Japan

Correction to: Environmental Management (2021) 68:353–365 10.1007/s00267-021-01497-4

The article “Outcome-Based Assessment of the Payment for Mountain Agriculture: A Community-Based Approach to Countering Land Abandonment in Japan” written by Kikuko Shoyama, Maiko Nishi, Shizuka Hashimoto, Osamu Saito was originally published Online First without Open Access. After publication in volume 68, issue 3, page 353-365 the author decided to opt for Open Choice and to make the article an Open Access publication. Open access publication was funded by the Japan Society for the Promotion of Science (JPJSBP120195008: Sustainable management of commons in socio-ecological production landscapes in Slovenia and Japan). Therefore, the copyright of the article has been changed to © The Author(s) 2022 and the article is forthwith distributed under the terms of the Creative Commons Attribution 4.0 International License, which permits use, sharing, adaptation, distribution and reproduction in any medium or format, as long as you give appropriate credit to the original author(s) and the source, provide a link to the Creative Commons licence, and indicate if changes were made. The images or other third party material in this article are included in the article’s Creative Commons licence, unless indicated otherwise in a credit line to the material. If material is not included in the article’s Creative Commons licence and your intended use is not permitted by statutory regulation or exceeds the permitted use, you will need to obtain permission directly from the copyright holder. To view a copy of this licence, visit http://creativecommons.org/licenses/by/4.0.

The original article has been corrected.

